# TSFNet: Temporal-Spatial Fusion Network for Hybrid Brain-Computer Interface

**DOI:** 10.3390/s25196111

**Published:** 2025-10-03

**Authors:** Yan Zhang, Bo Yin, Xiaoyang Yuan

**Affiliations:** School of Electrical Engineering and Automation, Harbin Institute of Technology, Harbin 150001, China; 24s106194@stu.hit.edu.cn (B.Y.); yuanxiaoyang1998@outlook.com (X.Y.)

**Keywords:** hybrid brain-computer interface, electroencephalography, functional near-infrared spectroscopy, deep learning, multimodal fusion

## Abstract

Unimodal brain–computer interfaces (BCIs) often suffer from inherent limitations due to the characteristic of using single modalities. While hybrid BCIs combining electroencephalography (EEG) and functional near-infrared spectroscopy (fNIRS) offer complementary advantages, effectively integrating their spatiotemporal features remains a challenge due to inherent signal asynchrony. This study aims to develop a novel deep fusion network to achieve synergistic integration of EEG and fNIRS signals for improved classification performance across different tasks. We propose a novel Temporal-Spatial Fusion Network (TSFNet), which consists of two key sublayers: the EEG-fNIRS-guided Fusion (EFGF) layer and the Cross-Attention-based Feature Enhancement (CAFÉ) layer. The EFGF layer extracts temporal features from EEG and spatial features from fNIRS to generate a hybrid attention map, which is utilized to achieve more effective and complementary integration of spatiotemporal information. The CAFÉ layer enables bidirectional interaction between fNIRS and fusion features via a cross-attention mechanism, which enhances the fusion features and selectively filters informative fNIRS representations. Through the two sublayers, TSFNet achieves deep fusion of multimodal features. Finally, TSFNet is evaluated on motor imagery (MI), mental arithmetic (MA), and word generation (WG) classification tasks. Experimental results demonstrate that TSFNet achieves superior classification performance, with average accuracies of 70.18% for MI, 86.26% for MA, and 81.13% for WG, outperforming existing state-of-the-art multimodal algorithms. These findings suggest that TSFNet provides an effective solution for spatiotemporal feature fusion in hybrid BCIs, with potential applications in real-world BCI systems.

## 1. Introduction

The brain–computer interface (BCI) represents a sophisticated amalgamation of software and hardware that facilitates the translation of users’ neural activity or intentions into commands for external devices [1,2]. Recently, various neurophysiological signals have been employed in BCI systems for analytical purposes, including electroencephalography (EEG) [3,4], functional near-infrared spectroscopy (fNIRS) [5], magnetoencephalography (MEG) [6,7], functional magnetic resonance imaging (fMRI) [8,9], and electrooculography (EOG) [10,11]. While fMRI and MEG provide superior spatial resolution, their complex acquisition requirements render them impractical for portable or real-time applications. EOG is straightforward to obtain but is highly vulnerable to noise interference. In contrast, EEG and fNIRS signals are relatively easy to acquire, and their artifacts can be more effectively removed. Consequently, EEG and fNIRS are regarded as more appropriate modalities for practical BCI implementations.

EEG and fNIRS offer complementary advantages due to their distinct focuses on different physiological activities of the brain. EEG is a non-invasive electrophysiological technique that quantifies voltage fluctuations resulting from ionic currents in neuronal activity, whereas fNIRS utilizes near-infrared light to assess variations in the concentrations of oxygenated and deoxygenated hemoglobin (HbO and HbR) [12,13]. EEG is characterized by its exceptional temporal resolution, enabling the detection of immediate brain activity responses; however, it is prone to interference from artifacts related to ocular movements, cardiac activity, and other extraneous factors [14]. Conversely, fNIRS offers enhanced spatial resolution and demonstrates considerable resilience to various forms of noise, although it is limited by a delayed response time [15]. Consequently, a dual-modal brain–computer interface (BCI) that integrates both EEG and fNIRS is likely to yield superior performance compared to a BCI that relies solely on either modality.

In hybrid BCI systems, it is crucial yet challenging to effectively leverage the complementary advantages offered by EEG and fNIRS, which makes effective multimodal fusion a widely researched topic [16,17,18,19,20]. Existing studies can be categorized into two principal types: feature-level fusion and decision-level fusion methods [21,22]. Feature-level fusion primarily refers to the integration of features during the early and mid-stages of the model [23]. For instance, Sun et al. proposed a pth-order polynomial fusion approach that achieves deep feature integration through multiple outer products of EEG and fNIRS feature vectors [24]. A novel framework that uses convolutional neural networks to generate and fuse multi-view spatiotemporal representations has been proposed, which can effectively integrate EEG and fNIRS features to achieve robust classification [25]. Liu et al. achieved precise temporal and spatial alignment between EEG and fNIRS features through an fNIRS-guided spatial alignment layer and an EEG-guided temporal alignment layer [26]. Arif et al. designed a deep learning network to effectively fuse well-extracted features from both modalities [27]. Gao et al. proposed a method for low-dimensional feature extraction from fNIRS based on an EEG-informed general linear model (GLM) analysis, which integrates mixed features obtained from the CSP features extracted from different frequency bands of fNIRS and EEG [28]. A method applying the Tunable Q-factor Wavelet Transform (TQWT) to EEG and fNIRS has been proposed, which selectively fuses the extracted relevant features to enhance the quality of multimodal representations [29]. Feature-level fusion allows for deep interaction between features from different modalities by leveraging their correlations. However, asynchronous characteristics in temporal and spatial domains between modalities remain a major obstacle to effective fusion at this level [30,31].

Decision-level fusion, in contrast, integrates the probabilistic outputs of different modalities to improve classification performance by exploiting their complementarity [32]. Qin et al. proposed an adaptive weight-based decision fusion method that performs a weighted sum based on the predicted scores from EEG, fNIRS, and fusion feature outputs [33]. Rabbani et al. employed maximum likelihood estimation to combine decision scores from multiple models and analyzed the fusion performance from a statistical perspective [34]. Nia et al. conducted an in-depth comparison of three voting-based decision fusion methods, highlighting their utility in integrating EEG and fNIRS at the decision level [32]. Si et al. proposed a decision fusion method based on dual-stream cross-attention, which integrates the outputs of dual-stream cross-attention through weighted summation to enhance decision robustness [35]. Decision-level fusion provides a statistical interpretation of multimodal complementarity and offers a flexible direction for fusion strategies. This approach allows dynamic adjustment of the outputs without modifying the model structure, providing a straightforward method for multimodal integration. However, it is important to note that changes in feature extraction significantly affect the performance of decision-level fusion [36].

Another challenge in EEG-fNIRS hybrid BCIs lies in determining which modality should dominate the fusion process. Although EEG and fNIRS are complementary, their temporal and spatial asynchrony can lead to misalignment in feature fusion, ultimately degrading classification performance. To address this issue, one modality may need to guide the alignment of the other to enable effective feature fusion [21,30,37]. For example, Li et al. proposed an EEG-informed fNIRS GLM framework in which EEG rhythms guide feature alignment within specific fNIRS frequency bands [38]. Kwak et al. developed a fNIRS-guided attention network (FGANet) that uses spatial attention features extracted from fNIRS to guide spatial alignment of EEG features [39]. Xu et al. proposed a novel EEG-fNIRS deep fusion network (EFDFNet), where fNIRS features guide the disentanglement of EEG features, enhancing the quality of the fused representation [40]. In addition to using EEG or fNIRS as the dominant modality individually, some approaches consider parallel guidance from both. For instance, Wang et al. captured the temporal features of EEG and the spatial information of fNIRS through a dual-modal parallel network and fused them to achieve interaction of spatiotemporal features [41]. Qiu et al. used an Atomic Search Optimization (ASO) algorithm to select time-frequency features extracted from both EEG and fNIRS, enabling the matching of multimodal features [42]. Despite the variety of fusion paradigms, the ultimate goal of EEG-fNIRS hybrid BCIs is to achieve synchronization between modalities to enhance feature correlation and complementarity [37,43].

To utilize complementary advantages between EEG and fNIRS signals, an effective multimodal fusion method is essential. Therefore, a novel deep learning-based fusion approach, termed the Temporal-Spatial Fusion Network (TSFNet), is proposed. The network consists of two sub-layers: the EEG-fNIRS-guided Fusion (EFGF) layer and the Cross-attention-based Feature Enhancement (CAFÉ) layer. The EFGF layer utilizes hybrid attention maps to perform feature fusion and extraction. The generation of a hybrid attention map, explicitly guided by the temporal features of EEG and the spatial features of fNIRS, will lead to a more effective and complementary integration of spatiotemporal information than feature concatenation or simple late fusion, thereby significantly improving classification performance. The CAFÉ layer employs a bidirectional cross-attention mechanism as its core structure. After an initial fusion, facilitating bidirectional interaction between the fusion features and the original fNIRS features via a cross-attention mechanism will further refine the feature representations, selectively enhancing informative components and suppressing noise or redundant information, which is expected to provide an additional performance boost. Subsequently, the outputs from three branches of the network are combined using learnable weights to generate the final prediction. By integrating feature-level and decision-level fusion, TSFNet fully exploits the complementary characteristics of both signals based on a parallel EEG-fNIRS dominant fusion strategy. Comprehensive comparative experiments are conducted on two publicly available datasets containing different tasks, demonstrating the superior performance of TSFNet.

The main contributions of this work are summarized as follows:

- A novel algorithm for hybrid BCI called TSFNet is proposed to enable effective complementarity between EEG and fNIRS signals, thereby enhancing the classification performance in subject-dependent cross-session classification experiments.
- An EEG-fNIRS-guided fusion layer is designed to perform a more effective feature fusion based on EEG-guided temporal feature learning and fNIRS-guided spatial feature learning, where a hybrid attention map is generated for complementary integration of spatiotemporal information.
- A cross-attention-based feature enhancement layer is introduced, which enables multimodal feature representations to be further refined via cross-attention mechanism, selectively enhancing informative components and suppressing noise or redundant information.

## 2. Dataset

### 2.1. Dataset Description

All experiments and model evaluations conducted in this study are based on two publicly available benchmark datasets [44,45].

**Figure 1:**
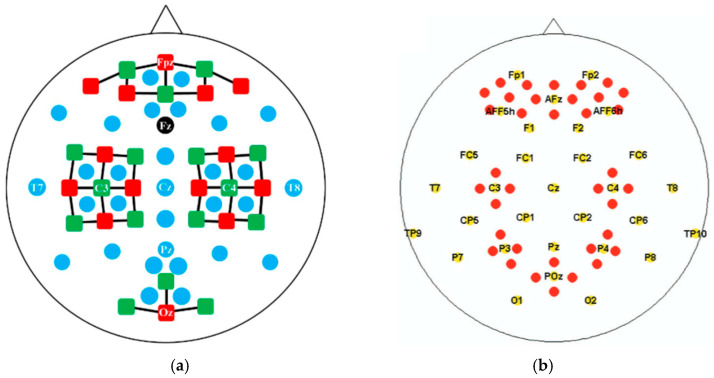
(**a**) The positions of EEG electrodes (blue and black circles) and the source-detector configuration for fNIRS (red squares) in dataset-A, which is sourced from [44]; (**b**) The positions of EEG electrodes (yellow circles) and fNIRS channels (red circles) in dataset-B, which is sourced from [45].

The multi-task Dataset A [44] includes EEG and fNIRS recordings from 29 healthy subjects (14 males and 15 females), consisting of 28 right-handed and 1 left-handed participants, with an average age of 28.5 ± 3.7 years. EEG signals were collected from 30 electrode channels at a sampling rate of 1000 Hz, while fNIRS signals were acquired from 36 physiological channels derived from 14 sources and 16 detectors at 12.5 Hz. The electrode positions for EEG and the source-detector configuration for fNIRS are shown in Figure 1a. As specified by the dataset provider, the EEG data were downsampled to 200 Hz and fNIRS data to 10 Hz. The dataset includes two task paradigms: motor imagery (MI) and mental arithmetic (MA). Each subject performed three sessions of both MI and MA tasks under identical experimental conditions. Each session consisted of a 1 min pre-rest period, 20 repeated task trials, and a 1 min post-rest period.

**Figure 2:**
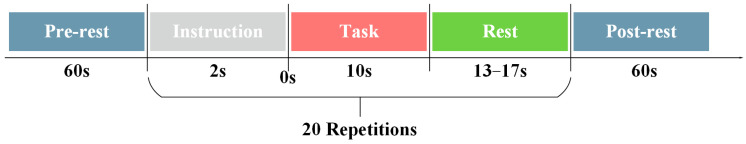
Schematic diagram of one session.

During the MI task, subjects were instructed to physically grasp a ball and simultaneously imagine forceful grasping with either the left or right hand as indicated by the cue. In the MA task, subjects performed both baseline (BL) and mental arithmetic experiments. The BL periods involved a low cognitive load resting state, while the MA trials required continuous mental subtraction of a one-digit number from a three-digit number (e.g., 264–267). A schematic diagram of one session is shown in Figure 2.

Dataset B for cognitive tasks [45] comprised EEG and fNIRS recordings from 26 healthy participants (9 males and 17 females) with an average age of 26.1 ± 3.5 years. EEG data were collected from 28 channels at 1000 Hz and later downsampled to 200 Hz, while fNIRS data were recorded from 36 physiological channels at 10.4 Hz and downsampled to 10 Hz. The electrode layout and source-detector configuration are shown in Figure 1b. The experimental paradigm in Dataset B involved a word generation (WG) task. During the WG trials, subjects were asked to rapidly generate as many non-repeating words as possible beginning with a given letter. In contrast, during the BL trials, subjects were asked to maintain relaxation while fixating on a cross to ensure low cognitive load. The session and trial design of the WG task is consistent with the MA task, as illustrated in Figure 2.

### 2.2. Data Preprocessing

For EEG data, a 6th-order zero-phase Butterworth bandpass filter with a passband of 0.5–50 Hz was applied to remove high-frequency and low-frequency noise [44]. The filtered EEG signals were then re-referenced using a common average reference (CAR). Finally, ocular artifacts were removed using independent component analysis (ICA) based on the EOG signals provided in the dataset [46].

For fNIRS data, changes in the concentrations of deoxygenated and oxygenated hemoglobin (HbR and HbO) were first computed using the modified Beer–Lambert law [47]. Subsequently, the HbR and HbO data were bandpass filtered within the 0.01–0.1 Hz range to remove physiological noise. In the end, baseline correction was performed by subtracting the average signal within the −5 to −2 s interval before each trial [44].

### 2.3. Three-Dimensional Tensor Transformation

To achieve spatial consistency between EEG and fNIRS modalities, the original 1D time series data were transformed into 3D tensors. For EEG data, according to [24,26,39,44], segments of 3 s in length were extracted using a sliding window from −2 to 10 s (instruction and task periods) with a step size of 1 s. Thus, each EEG sample had a dimension of 30 × 600 (channel × time). A 2D grid of size 16 × 16 was then generated by projecting the 3D positions of the EEG electrodes onto a 2D plane using azimuthal equidistant projection [48]. The EEG data from corresponding channels were mapped onto their projected locations, and missing values between electrode positions were interpolated using cubic spline interpolation [39]. In this way, each EEG sample was transformed into a 3D tensor, represented as $X^{eeg}\in {\mathbb{R}}^{16\times 16\times 600}$.

For fNIRS data, the same segmentation method was applied, resulting in multiple time windows of 3 s. Considering the inherent delay of the hemodynamic response in fNIRS compared to EEG, each EEG time window was paired with the current and subsequent 10 fNIRS segments [26]. As a result, each fNIRS sample had a shape of 11 × 36 × 30 × 2 (segment × channel × time × HbO & HbR). Finally, fNIRS data were transformed into 3D tensors using a method similar to EEG. However, the spatial projection was performed based on the positions of the physiological channels formed by source-detector pairs, rather than their actual positions on the scalp. Accordingly, each fNIRS sample was also converted into a 3D tensor, represented as $X^{fnirs}\in {\mathbb{R}}^{11\times 16\times 16\times 30\times 2}$.

## 3. Method

**Figure 3:**
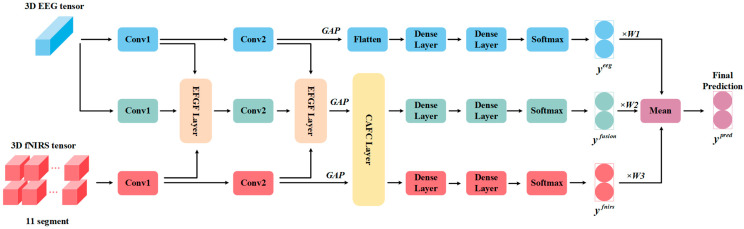
Framework of TSFNet.

We propose a novel Temporal-Spatial Fusion Network (TSFNet) to achieve deep integration of EEG–fNIRS temporal and spatial features. The overall framework of TSFNet is illustrated in Figure 3. TSFNet consists of four main components: a 3D convolutional neural network (CNN) structure, an EEG-fNIRS-guided fusion (EFGF) layer, a cross-attention-based feature enhancement (CAFE) layer, and a classifier. The 3D CNN structure captures spatiotemporal features from both EEG and fNIRS modalities and performs global feature aggregation through global average pooling (GAP). The EFGF layer fuses the spatiotemporal features from EEG and fNIRS utilizing a hybrid attention map. The CAFE layer further enhances the fusion features by selectively integrating supplementary information from the fNIRS modality. The classifier generates the final prediction scores by performing a weighted fusion of predictions from each modality branch.

### 3.1. Three-Dimensional CNN Structure

3D convolution has demonstrated superior performance in areas such as video recognition, medical images, and 3D object analysis, highlighting its effectiveness in extracting features from 3D data [49,50,51,52]. To capture informative temporal and spatial features from 3D EEG and fNIRS tensors, 3D convolutions are employed across all branches—EEG, fNIRS, and fusion. Each branch contains two 3D convolutional layers. Our experiments show that fewer layers fail to adequately extract spatiotemporal features, while additional layers do not significantly improve model performance. The detailed parameters of the 3D convolutional layers are summarized in Table 1.

For the EEG branch, given that the temporal dimension of the 3D EEG tensor is significantly larger than the spatial dimensions, convolutions with large strides and receptive fields are applied along the temporal axis, while smaller kernel sizes are used along the spatial axes to focus on localized spatial features. Adaptive padding, which is an approach for padding in Tensorflow, is employed to maintain a proportional change in the spatiotemporal feature map, thereby minimizing information loss. Each convolutional layer is followed by an exponential linear unit (ELU) activation function [53] to enhance nonlinearity. Batch normalization (BN) [54] and dropout [55] are applied to improve generalization and reduce overfitting. Finally, GAP is employed to aggregate temporal features and reduce the computational complexity of subsequent layers. The resulting EEG feature representation is denoted as $F^{eeg}\in {\mathbb{R}}^{4\times 4\times 32}$.

For the fNIRS branch, the feature extraction follows the same pipeline as the EEG branch. However, due to the shorter temporal length of fNIRS compared to EEG, and their shared spatial dimensions, small convolutional kernels are used for both spatial and temporal dimensions. After GAP, the resulting fNIRS feature representation is denoted as $F^{fNIRS}\in {\mathbb{R}}^{11\times 4\times 4\times 32}$.

For the fusion branch, since its input is entirely derived from the 3D EEG tensor, the same convolutional kernel parameters as in the EEG branch are adopted for 3D convolution. This strategy ensures the preservation of EEG features for better alignment with fNIRS data. Aside from the feature-level fusion performed through the EFGF layer after each convolutional layer, the rest of the processing pipeline remains consistent with the EEG branch. The extracted fusion feature is denoted as $F^{fusion}\in {\mathbb{R}}^{4\times 4\times 32}$.

### 3.2. EEG-fNIRS-Guided Fusion Layer

**Figure 4:**
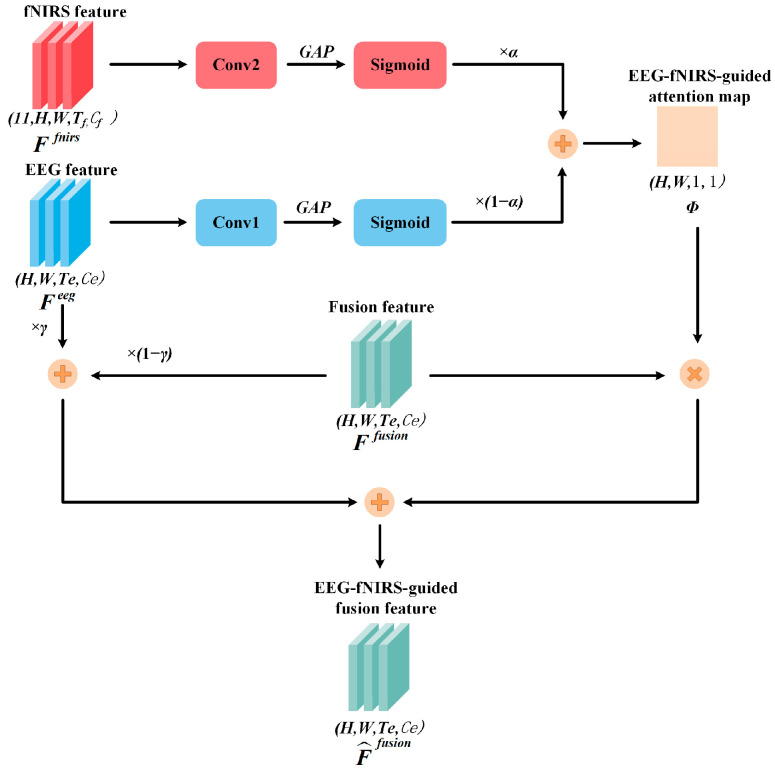
Structure of EFGF layer.

EEG provides superior temporal resolution, allowing for capturing rapid neural activity, while fNIRS offers excellent spatial resolution and sensitivity to deep brain regions. The effective integration of these two modalities enables the complementary strengths of each to be leveraged, resulting in representative spatiotemporal features. Inspired by [39], where spatial attention map extracted from fNIRS is utilized for spatial alignment with EEG, we propose a novel approach used in the EFGF layer, which can integrate temporal and spatial representations by a hybrid attention map. In the EFGF layer, an EEG-fNIRS-guided attention map is first generated by EEG temporal features and fNIRS spatial features using a gated fusion mechanism. This attention map is then applied to perform element-wise multiplication with the fusion branch features to obtain enhanced multimodal representations. In parallel, a residual connection with a trainable parameter integrates the original EEG features into the fusion process. The outputs from the two paths are summed to produce the final EEG-fNIRS-guided fusion feature. The structure of the EFGF layer is shown in Figure 4.

The inputs to the EFGF layer consist of features produced by the 3D convolutional layers of the EEG, fNIRS, and fusion branches. The output of EFGF layer is defined as:

(1) $${\hat{F}}^{fusion}=\gamma F^{eeg}+(1-\gamma )F^{fusion}+\Phi F^{fusion},$$

where $F^{eeg}$ and $F^{fusion}$ are the EEG and fusion features extracted by the 3D convolutional layers, respectively, $\gamma \in \left[0,1\right]$ is a trainable residual parameter, and the attention map Φ is computed as:

(2) $$\Phi =\alpha \sigma_{sig}(GAP(conv_{h,w,t}(F^{fnirs})))+(1-\alpha )\sigma_{sig}(GAP(conv_{h,w,t}(F^{eeg}))),$$

where $\alpha \in \left[0,1\right]$ is a trainable parameter, $\sigma_{\mathrm{sig}}$ denotes the Sigmoid function, GAP refers to global average pooling, $conv_{h,w,t}$ indicates the 3D convolution with one kernel size of (*h*, *w*, *t*), and $F^{fnirs}$ is the fNIRS feature input to the EFGF layer.

After GAP and convolution, both EEG and fNIRS features are transformed into feature maps of size (*h*, *w*, 1, 1). Due to their distinct properties, with EEG providing more temporal information and fNIRS providing more spatial information, the attention map captures comprehensive spatiotemporal relationships. Element-wise multiplication between the attention map and the original fusion features enables the integration of effective information. To mitigate loss of significant information due to unexpected low attention weights, a residual connection involving the EEG and fusion features is introduced, yielding the final deep-fused representation.

### 3.3. Cross-Attention-Based Feature Enhancement Layer

**Figure 5:**
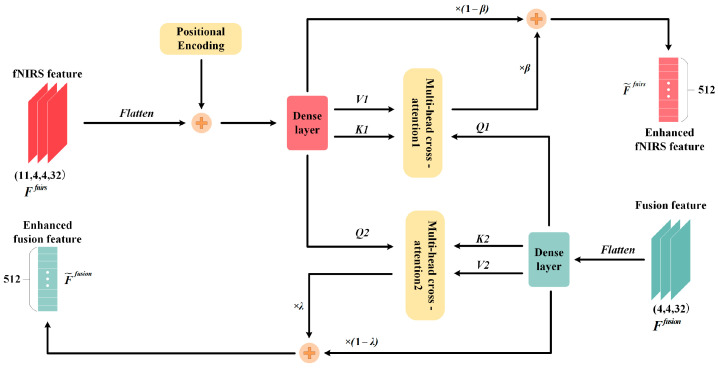
Structure of CAFE layer.

To further refine complementary information between the fusion and fNIRS features, the CAFE layer is introduced. The CAFE layer employs a dual-stream enhancement strategy based on cross-attention, allowing mutual guidance between fusion and fNIRS features to facilitate deeper spatiotemporal interaction. For the fusion features, the CAFE layer serves as an extension of the EFGF layer to recover any missing spatial information. For the fNIRS features, it enables selective feature refinement. Additionally, as the initial fusion features are derived exclusively from EEG features, the EEG feature is excluded from the CAFE layer for further enhancement and exchange. Instead, it is utilized as a fixed signal to ensure the stability of performance during subsequent decision-level fusion processes. The structure of the CAFE layer is illustrated in Figure 5.

The input to the CAFE layer consists of the fNIRS features $F^{fnirs}$ and fusion features $F^{fusion}$ extracted from the 3D CNN structure. These features are first flattened to facilitate subsequent computations. Since only certain segments of the 11 fNIRS time windows contain relevant information, learnable positional encodings are added to capture discriminative segments. Subsequently, both feature sets are projected into a lower-dimensional space to reduce computational complexity. Multi-head cross-attention mechanism is applied, with fusion features and fNIRS features alternately serving as the basis for computation, enabling bidirectional feature enhancement. Finally, gated fusion is employed to combine the enhanced and original features to improve robustness. Consequently, the outputs of the CAFE layer are the enhanced fusion features ${\tilde{F}}^{fusion}$ and the enhanced fNIRS features ${\tilde{F}}^{fnirs}$.

In the Transformer architectures, cross-attention typically involves three inputs: query (Q), key (K), and value (V). Q represents the features that need to attend to other inputs, K represents the features over which attention is calculated, and V represents the actual values of the input sequence. The attention weights are calculated by measuring the similarity between Q and K using dot-product operations, followed by a softmax function. These weights are then used to perform a weighted sum of V to produce the final output [51]. The process is defined as:

(3) $$Attention(Q,K,V)=\sigma_{soft}(\frac{QK^{T}}{\sqrt{d_{k}}})V,$$

where $\sigma_{soft}$ denotes the softmax function, and $d_{k}$ is the dimensionality of K.

In the CAFE layer, two cross-attention modules are utilized to enable bidirectional information flow between fNIRS and fused features. In cross-attention 1, fNIRS features attend to the fusion features—guided by learnable positional encodings—to extract the most relevant components aligned with the fusion representations. In cross-attention 2, information flows from fNIRS to the fusion features to enhance spatial characteristics. To enhance the model’s capacity for capturing complex relationships, multi-head attention is adopted, where Q, K, and V are divided into multiple heads. Each head attends to different subspaces or feature dimensions, preserving fine-grained variations across inputs. This multi-head mechanism facilitates thorough interaction between the spatiotemporal characteristics of the fusion and fNIRS features. The detailed configurations of the multi-head cross-attention mechanism follow the settings in [26].

### 3.4. Classifier

The classifier is designed to transform the final extracted features into predicted classification scores. The outputs of the CAFE layer, which contain enhanced fusion features ${\tilde{F}}^{fusion}$, enhanced fNIRS features ${\tilde{F}}^{fnirs}$ and the flattened EEG features $F_{flatten}^{eeg}$, are each passed through two dense layers followed by a softmax function, resulting in predictions $y^{fusion}$, $y^{fnirs}$, and $y^{eeg}$, respectively. A weighted averaging strategy is used to integrate these predictions into a final prediction score $y^{pred}$. It can be defined as:

(4) $$y^{pred}=Mean(w_{1}y^{eeg},w_{2}y^{fusion},w_{3}y^{fnirs}),$$

where $w_{1}$, $w_{2}$, $w_{3}$ are trainable weight coefficients obtained via a sigmoid function applied to initialized parameters, and *Mean* denotes the averaging operation.

This decision-level fusion approach integrates the prediction scores of the three branches, leveraging their complementary strengths to produce a more reliable final decision. By emphasizing higher-confidence predictions via learnable weights, and averaging across branches, this strategy not only ensures the score remains in a valid range but also enhances generalization performance.

### 3.5. Loss Function

To effectively train TSFNet, a comprehensive loss function that incorporates multiple components of the network is designed, including the classification loss *L_class_*, the EFGF regularization loss *L_efgf_*, and the CAFE regularization loss *L_cafe_*. The total loss *L* is defined as:

(5) $$L=L_{class}+L_{efgf}+L_{cafe}$$

#### 3.5.1. Classification Loss

Since the decision-level fusion employed in our model integrates the prediction scores from the EEG branch, the fNIRS branch, and the fusion branch, the loss function must also account for the performance of each individual branch as well as the final decision output. Therefore, the overall classification loss *L_class_* consists of four components: the classification loss of the EEG branch *L_eeg_*, the classification loss of the fNIRS branch *L_fnirs_*, the classification loss of the fusion branch *L_fusion_*, and the classification loss of the final decision output *L_pred_*. The overall classification loss is defined as:

(6) $$L_{class}=L_{pred}+\lambda_{eeg}L_{eeg}+\lambda_{fnirs}L_{fnirs}+L_{fusion},$$

where $\lambda_{eeg}$ and $\lambda_{fnirs}$ are classification loss parameters.

Classification loss serves as a key metric in supervised learning to quantify the discrepancy between predicted results and ground truth labels. To evaluate this discrepancy in the binary classification task, the cross-entropy loss function is adopted for each branch and the final prediction output. The corresponding formulations are as follows:

(7) $$L_{eeg}=-\frac{1}{N}\sum_{i=1}^{N}y_{i}log(y^{eeg}(X_{i}^{eeg})),$$

where *y_i_* denotes the ground truth label of the *i*-th input, $y^{eeg}(X_{i}^{eeg})$ represents the prediction scores of the *i*-th input tensor $X_{i}^{eeg}$ from the EEG branch, and *N* is the total number of samples.

(8) $$L_{fnirs}=-\frac{1}{N}\sum_{i=1}^{N}y_{i}log(y^{fnirs}(X_{i}^{fnirs})),$$

where $y^{fnirs}(X_{i}^{fnirs})$ represents the prediction scores of the *i*-th input tensor $X_{i}^{fnirs}$ from the fNIRS branch.

(9) $$L_{fusion}=-\frac{1}{N}\sum_{i=1}^{N}y_{i}log(y^{fusion}(X_{i}^{fnirs},X_{i}^{eeg})),$$

where $y^{fusion}(X_{i}^{fnirs},X_{i}^{eeg})$ represents the prediction scores of the *i*-th input tensor $\left(X_{i}^{fnirs},X_{i}^{eeg}\right)$ from the fusion branch.

(10) $$L_{pred}=-\frac{1}{N}\sum_{i=1}^{N}y_{i}log(y^{pred}({y_{i}}^{eeg},{y_{i}}^{fnirs},{y_{i}}^{fusion})),$$

where $\left(y^{pred}({y_{i}}^{eeg},{y_{i}}^{fnirs},{y_{i}}^{fusion}\right)$ represents the final prediction scores of the *i*-th input $\left({y_{i}}^{eeg},{y_{i}}^{fnirs},{y_{i}}^{fusion}\right)$.

#### 3.5.2. EFGF Regularization Loss

The EFGF layer is designed to effectively integrate the spatiotemporal features of EEG and fNIRS, leveraging their complementary characteristics. Consequently, the Pearson correlation coefficient (PCC) is employed to quantify the spatiotemporal correlation between the two modalities [38]. The EFGF layer is trained to maximize the PCC in order to enhance inter-modal feature correlation. The PCC is defined as:

(11) $$\mathrm{PCC}(U,V)=\frac{\sum_{i=1}^{N}\sum_{j=1}^{M}\left(u_{i,j}-\bar{u})(v_{i,j}-\bar{v}\right)}{\sqrt{\sum_{i=1}^{N}\sum_{j=1}^{M}\left(u_{i,j}-\bar{u}\right)}\sqrt{\sum_{i=1}^{N}\sum_{j=1}^{M}\left(v_{i,j}-\bar{v}\right)}},$$

where $\bar{u}$ and $\bar{v}$ denote the mean values of matrices *U* and *V*, respectively. Given that the PCC ranges from −1 to 1, the regularization loss for the EFGF layer is defined as:

(12) $$L_{efgf}=1-\frac{1}{2N}\sum_{i=1}^{N}\sum_{j=1}^{2}\left|PCC(\bar{F_{i,j}^{eeg}},\Phi_{i,j})\right|,$$

where $\bar{F_{i,j}^{eeg}}$ and $\Phi_{i,j}$ denote the average-pooled EEG feature map and the EEG-fNIRS-guided attention map of the *j*-th EFGF layer for the *i*-th input, respectively.

#### 3.5.3. CAFE Regularization Loss

The CAFE layer is constructed to enhance the fusion features and selectively extract informative fNIRS features through cross-attention. Similarly to the EFGF layer, PCC is employed to measure the correlation between the enhanced fusion features and the effective fNIRS features. The regularization loss for the CAFE layer is calculated as:

(13) $$L_{cafe}=1-\frac{1}{N}\sum_{i=1}^{N}\left|\mathrm{PCC}({{\tilde{F}}_{i}}^{fnirs},{{\tilde{F}}_{i}}^{fusion})\right|,$$

where ${{\tilde{F}}_{i}}^{fnirs}$ and ${{\tilde{F}}_{i}}^{fusion}$ denote the effective fNIRS features and the enhanced fusion features corresponding to the *i*-th input, respectively.

### 3.6. Experimental Setup

To investigate the cross-session variability of physiological features for individual subjects under the same task, the performance of TSFNet in subject-dependent cross-session classification was evaluated utilizing the two benchmark datasets described in Section 2. Due to each of the three tasks contained in the benchmark datasets includes three sessions, a Hold-Out evaluation was conducted for all three sessions of each task. In the Hold-Out analysis, one of the three sessions was selected in turn as the test set, while the remaining two sessions were utilized as the training set. This procedure was repeated until each session had been used as the test set. To improve training efficiency and reduce unnecessary training epochs, a two-stage training strategy [26] was employed. In the first stage, the training set was randomly split into a first-stage training subset and a validation set in a 4:1 ratio. Training in the first stage was terminated when the validation accuracy showed no improvement for 50 consecutive epochs. To prevent excessive training, the maximum number of training epochs in the first stage was set to 300. After the termination or completion of the first-stage training, the network parameters corresponding to the highest validation accuracy were restored. In the second stage, the entire training set was used for model training. This stage was stopped when the training loss dropped below that of the first stage. Similarly to the first stage, a maximum of 200 training epochs was allowed. The trained model was then evaluated on the test set. The average of test accuracy across three sessions was reported as the final performance metric. To mitigate the impact of chance agreement and better reflect the true effectiveness of the classifier, the Kappa coefficient was introduced as an additional evaluation metric. The Kappa coefficient is calculated as follows:

(14) $$Kappa=\frac{p_{0}-p_{e}}{1-p_{e}},$$

where $p_{0}$ denotes the observed accuracy, and $p_{e}$ denotes the expected accuracy by random chance.

The Adam optimizer was employed with a learning rate of 0.001 to update the network parameters. All trainable residual parameters were initialized to zero. Network hyperparameters and loss parameters $\lambda_{eeg}$ and $\lambda_{fnirs}$ were optimized through a parameter tuning process. During this tuning phase, the same training and evaluation procedures described above were followed. Finally, the optimal network configuration is determined as presented in Table 1 and the best-performing loss weights $\lambda_{eeg}$ and $\lambda_{fnirs}$ are both set to 0.2.

## 4. Results

### 4.1. Performance Comparison

#### 4.1.1. Overall Performance

The performance of TSFNet was compared with several state-of-the-art algorithms, as summarized in Table 2. Among all methods, STA-Net was re-implemented and its hyperparameters were set according to the optimal configuration reported in the original publication. The results of other algorithms were obtained from [26]. To ensure a fair comparison, all methods were trained and evaluated using the same strategy and procedures.

**Figure 6:**
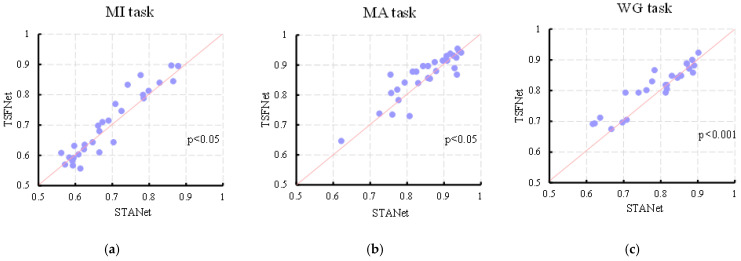
Scatter plots of classification accuracy of each subject for the MI (**a**), MA (**b**), and WG (**c**) tasks, with the horizontal axis representing accuracy of STA-Net and the vertical axis denoting accuracy of TSFNet.

Among the compared approaches, STA-Net achieves the best overall performance prior to the introduction of TSFNet. To verify that the improvement achieved by TSFNet over STA-Net was statistically significant, a paired *t*-test was conducted on the overall average classification accuracy. As shown in Table 2 and Figure 6, TSFNet outperformed STA-Net in all three tasks with statistical significance. Specifically, for the MI, MA, and WG tasks, TSFNet achieved improvements of 1.15% (*p* < 0.05), 1.49% (*p* < 0.05), and 2.14% (*p* < 0.001) in average classification accuracy, respectively. Corresponding increases in Kappa values were 2%, 3%, and 4%, respectively. The results indicate that TSFNet demonstrates superior classification performance compared to the other methods. In addition, the classification accuracy of each individual subject is reported in Table 3.

**Figure 7:**
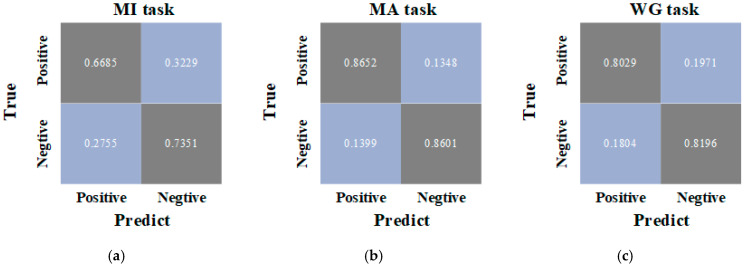
Confusion matrix of TSFNet for the MI (**a**), MA (**b**) and WG (**c**) tasks.

To illustrate the class-wise balance of TSFNet in the three binary classification tasks, confusion matrices were plotted, as shown in Figure 7. The confusion matrices indicate that TSFNet achieved balanced classification across the two classes, which aligns with the originally balanced distribution of labels. Moreover, the close alignment between precision and recall suggests that the excellent classification accuracy of TSFNet was not achieved at the expense of biased predictions.

#### 4.1.2. Performance Across Time Windows

**Figure 8:**
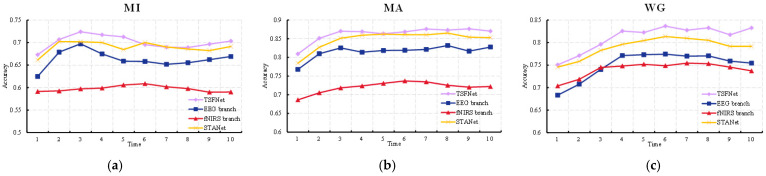
Average accuracy across all subjects for each time window for the MI (**a**), MA (**b**) and WG (**c**) tasks.

As described in Section 2, both EEG and fNIRS data were segmented into time windows of 3 s in length. To evaluate the classification performance of TSFNet across different time segments in the cross-session experiments, the classification accuracy of each sliding window was calculated. The average accuracy across all subjects for each time window is illustrated in Figure 8, where the *x*-axis denotes the right edge of each sliding window (e.g., “3” represents the 0–3 s window).

As shown in Figure 8, TSFNet consistently outperformed STA-Net in terms of average classification accuracy across nearly all time windows in the MI, MA, and WG tasks. This indicates that the performance improvement achieved by TSFNet is not restricted to specific time windows but is distributed across the entire time course. Additionally, the classification accuracy of the EEG and fNIRS branches within TSFNet for different time windows is also presented in the figure. For all tasks, the EEG branch reached its peak of accuracy in the time window with the right edge of 3 or 4 s, suggesting that brain activity was most prominent within the first few seconds of task initiation. In contrast, the fNIRS branch exhibited a delayed peak in accuracy, which is consistent with the hemodynamic lag responses.

### 4.2. Ablation Study

#### 4.2.1. Ablation on Sub-Layers of TSFNet

To evaluate the contribution of the EFGF and CAFE sub-layers to the overall performance of TSFNet, ablation experiments were conducted, as summarized in Table 4. In the MI task, eliminating the EFGF layer led to a 3.8% decrease in classification accuracy (*p* < 0.001), while the removal of the CAFE layer resulted in a 4.03% reduction (*p* < 0.001). For the MA task, removing the EFGF layer caused a 3.15% drop in accuracy (*p* < 0.001), and the exclusion of the CAFE layer led to a slight degradation in performance, with a 1.98% decrease (*p* < 0.001). In the WG task, accuracy declined by 1.88% and 2.95% (*p* < 0.001) when the EFGF and CAFE layers were removed, respectively. All results were verified by paired *t*-tests, indicating that both sub-layers contributed significantly to the model’s effectiveness from a statistical perspective.

**Figure 9:**
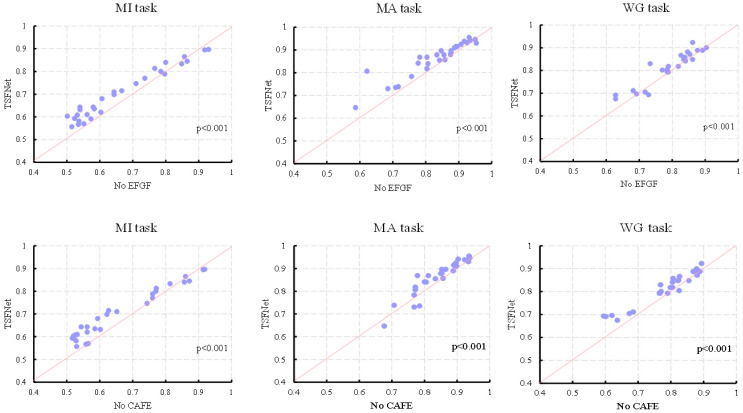
Scatter plots of classification accuracy of each subject for the MI, MA, and WG tasks, with the horizontal axis representing the accuracy after ablation and the vertical axis denoting accuracy of TSFNet.

To visually illustrate the influence of the two sub-layers on overall performance, scatter plots were generated showing classification accuracy for each subject under different ablation conditions, as depicted in Figure 9. It can be observed that the majority of the data points appear above the diagonal line when the EFGF layer is removed, while more points appear below the line after the removal of the CAFE layer. This suggests that the EFGF layer has a more pronounced impact on most subjects compared to the CAFE layer. Although the absence of the EFGF layer in the WG task resulted in a smaller reduction in average accuracy compared to the CAFE layer, this was due to significant improvements in accuracy for a subset of subjects after applying the CAFE layer. These improvements in performance demonstrate the enhancement effect of the CAFE layer on feature representation.

**Figure 10:**
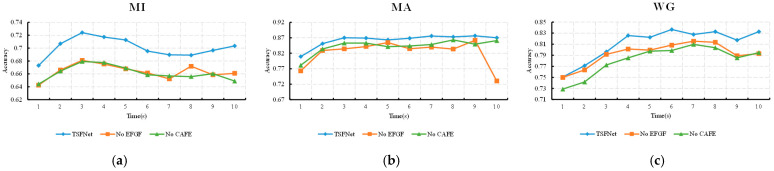
Line plots of average classification accuracy across different time windows under var-ious ablation conditions for the MI (**a**), MA (**b**) and WG (**c**) tasks.

To explore the influence of each sub-layer across time segments, line plots of average classification accuracy across different time windows under various ablation conditions were plotted, as shown in Figure 10. When only the EFGF layer was retained, the trend of the accuracy curve remained closely aligned with that of the original TSFNet. This highlights the influence of the EEG-guided temporal weights in the EFGF layer on classification performance across time segments. In contrast, when only the CAFE layer was present, a noticeable increase in accuracy was observed during the latter time periods, which can be attributed to the enhancement of fusion features by the fNIRS-derived information within the CAFE layer. The joint effect of both sub-layers contributes to the superior classification performance of TSFNet across all time windows.

#### 4.2.2. Ablation on Regularization Loss

Since the Pearson correlation coefficient was employed as the regularization loss function for each sublayer, an ablation study was conducted to investigate the effectiveness of the regularization loss function in the model. The results are presented in Table 5. The removal of *L_efgf_* led to a significant decrease in accuracy across all three tasks (*p* < 0.01), with a particularly notable reduction of 5.19% observed for the WG task. This indicates that when the EFGF layer is not sufficiently trained, its presence adversely affects model performance. This phenomenon is likely attributed to the numerous trainable gated fusion parameters and residual connection parameters contained within the EFGF layer. Similarly, the elimination of *L_cafe_* also resulted in a decline in accuracy (*p* < 0.01), though the magnitude of reduction was relatively modest. This may be due to the limited impact of the CAFE layer on classification performance. Collectively, these findings demonstrate that the regularization loss functions of the sub-layers play a critical role in guiding feature fusion toward the desired objective through the regulation of gated fusion and residual connection parameters, thereby significantly contributing to overall model performance.

### 4.3. Computational Complexity of TSFNet

To investigate the model complexity of TSFNet and its sub-layers, we conducted a detailed analysis of the number of parameters, training time, and inference time during testing. The results are summarized in Table 6. Specifically, the experiments were performed on an NVIDIA GeForce RTX 3090 GPU platform (NVIDIA Corporation, Santa Clara, CA, USA). Training time refers to the average time required to complete a single training epoch, while inference time denotes the average duration for processing a single batch of samples during testing. As shown in Table 6, the slight increases in the number of parameters, training time, and inference time of TSFNet are acceptable considering its significant performance improvements. Moreover, removing the CAFE layer leads to a substantial reduction in the number of parameters, accompanied by minor decreases in both training and inference time. This indicates that although the CAFE layer contains a large number of parameters, it requires only minimal additional time for adequate training and inference. These findings validate that the relationship between the number of parameters and the training or inference time should not be oversimplified or generalized.

## 5. Limitations and Future Work

In this study, a novel deep learning framework for hybrid BCI, called the Tem-poral-spatial Fusion Network (TSFNet), is proposed to fully exploit the complementary advantages of EEG and fNIRS signals. TSFNet employs three parallel branches and multi-stage information interaction across sub-layers to progressively integrate and enhance multimodal features. Experimental results have confirmed that TSFNet demonstrated superior performance across three tasks on two public datasets. Compared to other state-of-the-art hybrid BCI methods, TSFNet achieves higher mean accuracy and kappa values. Moreover, statistical analysis of performance over time shows that after an initial performance boost, TSFNet maintains high accuracy throughout most time segments. Notably, EEG signals demonstrates superior performance compared to fNIRS across all tasks. However, fNIRS exhibited greatly significant performance in the WG task. This observation suggests that fNIRS plays a more prominent role in cognitive tasks than in motor imagery tasks. These findings are consistent with conclusions reported in previous studies [13,15,22]. Specially, our study did not identify a single dominant modality across specific tasks that was statistically conclusive. Additionally, the ablation study provides critical insights for future hybrid BCI system design by validating the architectural choices of TSFNet. Our results demonstrate that both the EFGF and CAFE layers are indispensable components for achieving high performance across all three distinct tasks (MI, MA, WG). As detailed in Table 4, the consistent performance drop observed upon removing either layer indicates that effective spatiotemporal feature fusion and deep inter-modal feature enhancement are universally beneficial processes in EEG-fNIRS fusion, rather than being specific to a particular type of cognitive or motor task. This suggests that future hybrid BCI systems should prioritize integrating such dual mechanisms to ensure robust performance. The ablation study confirms that a successful fusion framework must simultaneously address the fundamental challenges of integration of temporal-spatial representations and bidirectional feature refinement.

Despite the remarkable performance, several limitations remain. First, due to the uncertain delay responses inherent in fNIRS signals, multiple time segments are included for feature selection, which substantially increase the computational cost. Second, the EFGF layer involves a number of residual connection and gating parameters, which are highly dependent on model training and lack clear interpretability. Third, although cross-session classification experiments and brief analyses are performed, the heterogeneity and consistency across sessions are not thoroughly investigated. In future work, efforts will be made to localize the most relevant fNIRS time segments during preprocessing, reducing the input size of fNIRS tensor. In addition, more interpretable parameters, such as weight maps derived directly from features, will be considered to replace the less meaningful trainable residual connection and gating parameters. A more in-depth analysis of the differences caused by cross-session classifications will also be conducted to better understand the impact of discontinuous tasks on brain activity.

## 6. Conclusions

A multi-branch hybrid BCI framework, called the Temporal-Spatial Fusion Network (TSFNet), is proposed in this study. Within TSFNet, the EFGF layer integrates EEG-guided temporal weights and fNIRS-guided spatial weights into an attention map, effectively utilizing the distinct spatiotemporal properties of the two modalities and achieving complementary fusion. The CAFE layer enhances and filters features by establishing across-modal correlations through cross-attention mechanisms. Comparative experiments demonstrate that TSFNet delivers superior performance across MI, MA, and WG tasks from two public datasets. Overall, the proposed model exhibits strong adaptability in EEG-fNIRS hybrid BCI applications.

## Figures and Tables

**Table 1:** Parameters of the 3D convolutional layers.

Branch	Layer	Kernel			Output
		Size	Stride	Channel	Dimension
EEG	Conv1	4 × 4 × 12	2 × 2 × 6	16	8 × 8 × 100 × 16
	Conv2	2 × 2 × 6	2 × 2 × 2	32	4 × 4 × 50 × 32
fNIRS	Conv1	4 × 4 × 6	2 × 2 × 2	16	11 × 8 × 8 × 15 × 16
	Conv2	2 × 2 × 3	2 × 2 × 2	32	11 × 4 × 4 × 8 × 32
Fusion	Conv1	4 × 4 × 12	2 × 2 × 6	16	8 × 8 × 100 × 16
	EFGF1 Conv1	2 × 2 × 6	1 × 1 × 1	1	8 × 8 × 100 × 1
	EFGF1 Conv2	2 × 2 × 3	1 × 1 × 1	1	11 × 8 × 8 × 15 × 1
	Conv2	2 × 2 × 6	2 × 2 × 2	32	4 × 4 × 50 × 32
	EFGF2 Conv1	2 × 2 × 3	1 × 1 × 1	1	4 × 4 × 50 × 1
	EFGF2 Conv2	2 × 2 × 3	1 × 1 × 1	1	11 × 4 × 4 × 8 × 1

**Table 2:** Overall performance of TSFNet and other state-of-the-art methods (mean ± std).

Methods	MI		MA		WG	
	Accuracy (%)	Kappa	Accuracy (%)	Kappa	Accuracy (%)	Kappa
FGANet [39]	64.95 ± 8.65	0.30 ± 0.17	76.58 ± 9.00	0.53 ± 0.18	71.33 ± 9.01	0.43 ± 0.18
EF-Net [27]	61.80 ± 8.04	0.24 ± 0.16	75.59 ± 9.55	0.51 ± 0.19	68.51 ± 9.30	0.37 ± 0.19
BiMNC [56]	67.25 ± 7.37	0.34 ± 0.15	82.36 ± 9.24	0.65 ± 0.18	76.86 ± 10.22	0.54 ± 0.20
M2NN [57]	66.21 ± 9.85	0.32 ± 0.20	82.72 ± 7.30	0.65 ± 0.15	76.07 ± 9.87	0.52 ± 0.20
EFMLNet [58]	66.92 ± 8.99	0.34 ± 0.18	83.14 ± 7.64	0.66 ± 0.15	77.49 ± 9.05	0.55 ± 0.18
E-FNet [59]	64.91 ± 7.51	0.30 ± 0.15	83.66 ± 7.87	0.67 ± 0.16	76.74 ± 9.89	0.53 ± 0.20
STA-Net [26]	69.03 ± 9.46	0.38 ± 0.20	84.77 ± 7.83	0.70 ± 0.16	78.99 ± 8.77	0.58 ± 0.18
TSFNet	**70.18 ± 10.84**	**0.40 ± 0.21**	**86.26 ± 7.45**	**0.73 ± 0.15**	**81.13 ± 7.18**	**0.62 ± 0.14**

The bold entries indicate the best values.

**Table 3:** Classification accuracy of each individual subject.

Subject Numbers	MI Accuracy (%)		MA Accuracy (%)		WG Accuracy (%)	
	TSFNet	STA-Net	TSFNet	STA-Net	TSFNet	STA-Net
01	**78.83**	78.50	**91.50**	89.67	79.33	**81.33**
02	**80.00**	78.33	85.50	**86.17**	85.83	**88.67**
03	**81.33**	79.83	**93.17**	92.50	**88.83**	87.00
04	64.33	**70.33**	**91.00**	87.50	**81.83**	81.33
05	**59.33**	58.33	**81.83**	77.33	**81.83**	81.67
06	57.00	**57.17**	**93.83**	91.67	**69.33**	62.17
07	**63.50**	62.50	**88.00**	87.83	**86.67**	78.33
08	60.33	**60.83**	**95.50**	93.67	70.50	**70.83**
09	**89.67**	86.00	73.00	**80.67**	84.17	**84.50**
10	56.67	**59.33**	**89.67**	85.67	**79.33**	74.00
11	58.17	**59.17**	73.50	**76.00**	**71.17**	63.67
12	64.33	**64.67**	**80.67**	75.67	**84.83**	83.00
13	**68.00**	66.50	89.00	**92.83**	69.67	**69.67**
14	62.00	**62.33**	85.67	**85.67**	88.17	**89.00**
15	**71.50**	69.00	**78.33**	77.67	**67.50**	66.67
16	**74.67**	72.50	**73.83**	72.50	**92.33**	90.17
17	**77.00**	70.83	92.50	**93.33**	80.50	**81.67**
18	61.00	**66.50**	94.17	**94.67**	**80.17**	76.17
19	**86.50**	77.67	**84.17**	79.33	**83.00**	77.67
20	**60.83**	56.17	**64.67**	62.17	**79.33**	70.50
21	**71.00**	67.33	**89.67**	84.33	**69.17**	61.67
22	**63.17**	59.67	**87.83**	81.50	**90.00**	88.50
23	**83.33**	74.17	**84.00**	83.00	**88.83**	87.00
24	55.67	**61.33**	**91.50**	90.83	85.00	**85.33**
25	**84.00**	82.83	**86.83**	75.50	84.83	**85.50**
26	**89.50**	87.83	**94.67**	94.00	87.17	**87.67**
27	84.50	**86.50**	**87.83**	82.50		
28	59.17	**59.50**	86.83	**93.50**		
29	**69.83**	66.17	**93.00**	90.67		

The bold entries indicate the best values.

**Table 4:** Results of ablation study on sub-layers of TSFNet (mean ± std).

Task	Model	Accuracy (%)	Kappa
MI	No EFGF	66.38 ± 13.36	0.33 ± 0.27
	No CAFE	66.15 ± 13.46	0.33 ± 0.27
	TSFNet	**70.18 ± 10.84**	**0.40 ± 0.21**
MA	No EFGF	83.11 ± 9.40	0.66 ± 0.19
	No CAFE	84.28 ± 6.89	0.69 ± 0.14
	TSFNet	**86.26 ± 7.45**	**0.73 ± 0.15**
WG	No EFGF	79.25 ± 7.48	0.59 ± 0.15
	No CAFE	78.18 ± 9.01	0.56 ± 0.18
	TSFNet	**81.13 ± 7.18**	**0.62 ± 0.14**

The bold entries indicate the best values.

**Table 5:** Results of ablation study on regularization loss (mean ± std).

Task	Loss Function	Accuracy (%)	Kappa
MI	No *L_efgf_*	66.41 ± 10.80	0.33 ± 0.22
	No *L_cafe_*	66.56 ± 12.77	0.33 ± 0.26
	*L*	**70.18 ± 10.84**	**0.40 ± 0.21**
MA	No *L_efgf_*	83.25 ± 8.56	0.67 ± 0.17
	No *L_cafe_*	84.44 ± 7.79	0.69 ± 0.16
	*L*	**86.26 ± 7.45**	**0.73 ± 0.15**
WG	No *L_efgf_*	75.94 ± 9.55	0.52 ± 0.19
	No *L_cafe_*	79.22 ± 8.43	0.58 ± 0.17
	*L*	**81.13 ± 7.18**	**0.62 ± 0.14**

The bold entries indicate the best values.

**Table 6:** Computational complexity indicators of different models. T_train_ denotes the average training time per epoch across all training phases of the three tasks, T_infer_ refers to the average inference time per batch of samples across all testing phases of the three tasks.

Model	Parameters	T_train_ (ms)	T_infer_ (ms)
TSFNet	3,335,576	594.21	25.88
STA-Net	3,325,150	565.66	24.72
No EFGF	3,334,224	513.81	25.18
No CAFE	569,238	556.61	25.33

## Data Availability

This work uses public datasets, and the data links for dataset-A and dataset-B are https://doc.ml.tu-berlin.de/hBCI/ (accessed on 15 October 2024) and http://doc.ml.tu-berlin.de/simultaneous_EEG_NIRS/ (accessed on 9 April 2025).
